# Diagnostic performance of the FluoroType MTBDR assay for rifampicin and isoniazid resistance in routine laboratory setting

**DOI:** 10.4102/phcfm.v18i1.5034

**Published:** 2026-01-08

**Authors:** Ivy Rukasha, Kabelo G. Kaapu, Molebogeng R. Lekalakala-Mokaba

**Affiliations:** 1 Department of Pathology, School of Medicine, Faculty of Health Sciences, University of Limpopo, Polokwane, South Africa; 2 Department of Microbiology, National Health Laboratory Service, Polokwane, South Africa

**Keywords:** tuberculosis, drug resistance, molecular methods, rifampicin, isoniazid, multidrug resistance

## Abstract

**Background:**

Drug-resistant tuberculosis (DR-TB) continues to be a major public health threat, especially in high-burden settings like South Africa. Rapid and accurate diagnosis of resistance to rifampicin (RIF) and isoniazid (INH) is essential to ensure that patients receive the right treatment as early as possible. Current diagnostic tools, though effective, have limitations. This study addresses the urgent need for faster, reliable alternatives to improve DR-TB detection.

**Aim:**

The aim of this study is to assess the performance of the FluoroType MTBDR assay in detecting resistance to RIF and INH in a real-world diagnostic setting.

**Setting:**

The study was carried out at the National Health Laboratory Services (NHLS) Polokwane Laboratory, a routine laboratory in Limpopo province, South Africa.

**Methods:**

We tested 152 *Mycobacterium tuberculosis* (MTB) isolates collected from the 2023 laboratory repository using the FluoroType MTBDR version 2 assay. These results were compared with those from two established methods: GenoType MTBDRplus and Xpert MTB/RIF Ultra. Whole genome sequencing (WGS) was used to resolve any discrepancies, serving as the reference standard. Diagnostic accuracy was evaluated using sensitivity, specificity and predictive values.

**Results:**

Of the 152 isolates, 65% were drug-resistant. FluoroType MTBDR showed excellent performance – 100% specificity and 96% sensitivity for RIF resistance and 91% sensitivity and 99% specificity for INH resistance – competing with the GenoType MTBDRplus.

**Conclusion:**

FluoroType MTBDR offers a reliable, rapid alternative for detecting DR-TB, with the potential to improve timely diagnosis and treatment.

**Contribution:**

This study highlights the FluoroType MTBDR assay as a valuable diagnostic tool for routine use, contributing to improved TB control strategies, especially in resource-limited, high-burden settings, consistent with the journal’s scope.

## Introduction

Tuberculosis (TB), caused by *Mycobacterium tuberculosis* (MTB), remains a significant public health concern, with a global estimate of nearly two billion people – about one-fourth of the world’s population – is infected.^1^ To combat this epidemic, the World Health Organization (WHO) has implemented various strategies over the years, which include the ‘Directly Observed Therapy Strategy’ (DOTS) (1994–2005), the ‘Stop TB Stra*tegy’ (2006–2015) (Stop TB Partnership, 2006) and the current ‘End TB Strategy’ (2016–2030).^2^ However, despite the comprehensive WHO interventions, TB detection remains insufficient, with only two out of every three patients with TB detected.^1^ The continuous underdiagnosis of TB is a major hurdle for the eradication of the disease.

One solution to close the diagnostic gap for the diagnosis of TB and drug resistance is the use of centralised high-throughput platforms for the detection of *Mycobacterium tuberculosis* complex (MTBC) and molecular drug susceptibility testing. For the disease to be properly managed, prevent the spread and guarantee the early beginning of suitable treatment regimens, a quick and accurate laboratory diagnosis of the sensitivity and resistance of TB drugs is essential.^3^

The WHO has so far approved a number of polymerase chain reaction (PCR)-based assays for the detection of drug-resistant tuberculosis (DR-TB) testing, which includes MTBDRplus (Hain Lifescience, Nehren, Germany), the BD MAX MDR-TB assay and Xpert MTB/RIF Ultra (Cepheid, Sunnyvale, CA, United States [US]). The primary limitation of the standard Xpert MTB/RIF Ultra assay is that it only detects rifampicin (RIF) resistance, necessitating additional tests to confirm isoniazid (INH) and second-line drug resistance, which increases costs. Similarly, the LPA assay requires multiple processing steps, demanding skilled personnel and increasing operational costs. In addition, the LPA involves an extensive number of manual steps for both preparation and interpretation of results.^4^ Therefore, there is a critical need for a diagnostic assay that bridges this gap by enabling the simultaneous detection of both RIF and INH resistance while streamlining the testing process for greater efficiency. The BD MAX MDR-TB assay shows ease of use, speed and broad applicability; however, the assay has also been shown to have low sensitivity and thus may have lower use in detecting samples with low levels of bacilli such as sputum from children, human immunodeficiency viruses (HIV)-infected individuals and smear-negative cases.^5^ The FluoroType MTBDR assay has emerged as a molecular diagnostic tool for detecting MDR-TB. The Fluorotype MTBDR assay has consistently shown high sensitivity and the ability to detect MDR-TB in challenging samples with low bacilli.^6^

The FluoroType MTBDR assay targets INH and RIF resistance-associated genes and employs advanced linear-after-the-exponential PCR technology for detection.^7^ The assay generates melting curves as readouts, with specific curve shapes indicative of wild-type or mutant alleles. Unlike the GenoType MTBDRplus, the FluoroType MTBDR assay utilises a fully automated, closed system that can process up to 95 samples in a single batch, reducing contamination risks and human errors. Moreover, its automated deoxyribonucleic acid (DNA) extraction and interpretation capabilities streamline the diagnostic process and ensure faster, more accurate results.^7^

The FluoroType MTBDR assay has been evaluated in several countries, including Germany and South Africa.^7,8^ However, these evaluations have primarily taken place in controlled research settings. Its performance under routine laboratory conditions, particularly in rural laboratories, remains insufficiently explored. Therefore, the aim of the study is to assess the diagnostic accuracy of the FluoroType MTBDR assay in a routine microbiology laboratory setting and to evaluate its potential as a reliable efficient alternative to existing diagnostic methods.

## Research methods and design

### Study design

This study employed a quantitative diagnostic accuracy design to evaluate the performance of the FluoroType MTBDR assay for detecting MDR-TB in a routine laboratory setting. The quantitative methodology incorporated statistical analyses of diagnostic performance metrics, such as sensitivity, specificity, positive predictive value (PPV) and negative predictive value (NPV), to assess the precision of FluoroType MTBDR. The assay’s performance was compared to two established diagnostic tools: the GenoType MTBDRplus line probe assay (LPA), recognised as the conventional gold standard for molecular drug susceptibility testing, and the Xpert MTB/RIF Ultra assay, widely implemented in routine diagnostics for rapid TB detection. By benchmarking FluoroType MTBDR against these reference methods, the study provides a comprehensive evaluation of its diagnostic efficacy and potential utility as an alternative molecular diagnostic tool for MDR-TB detection.

### Study setting

The study was conducted at the National Health Laboratory Services (NHLS) Polokwane laboratory, a routine diagnostic laboratory located in Limpopo, South Africa. This laboratory provides specialised diagnostic services for both drug-sensitive and DR-TB across the province, serving the five districts of Limpopo: Waterberg, Capricorn, Sekhukhune, Vhembe and Mopani. As a referral centre, the laboratory handles complex diagnostic workflows and maintains a comprehensive TB isolate repository, making it a critical hub for TB diagnostics in the region. Limpopo itself is a predominantly rural province, where many communities face barriers to accessing healthcare services, underscoring the importance of timely and accurate TB diagnosis. The NHLS Polokwane Laboratory’s infrastructure and its central role in regional TB control made it an ideal setting to evaluate the performance of the FluoroType MTBDR assay under routine diagnostic conditions.

### Study population

The study population comprised 152 previously collected MTB isolates from patients across Limpopo province, South Africa, representing a diverse clinical mix of drug-resistant and drug-sensitive TB cases. These isolates, drawn from a routine diagnostic setting, reflect real-world conditions in a high TB-burden, resource-limited environment.

### Study samples

The study samples used were stored in the NHLS TB repository that had previously been tested for MDR-TB using the GenoType MTBDRplus LPA during the diagnostic period January 2023 – December 2023. A subset of these samples also had prior whole genome sequencing (WGS) results. These cryopreserved samples, originally collected before the study commenced, were retrospectively analysed to address the study objectives. The results of the GenoType MTBDRplus assay, obtained during routine diagnostic tests, were retrieved and used as a reference for comparison.

### Sampling technique

The study used simple random sampling from a pool of eligible stored isolates (*n* = 300) in the NHLS TB repository for the year 2023. Each sample was assigned a numeric code, and a computerised random number generator was used to select 152 samples without replacement. This ensured that every sample had an equal chance of being included and reduced selection bias. The final sample represented a balance of RIF-resistant and RIF-sensitive isolates as determined by prior MTBDRplus testing. The sample size was determined based on the number of high-quality, well-characterised isolates available with complete reference data, rather than a formal statistical power calculation.

### Subculture and quality control

The cryopreserved isolates were sub-cultured in Mycobacterial Growth Indicator Tubes (MGIT) culture medium using growth supplement and PANTA (BD, Franklin Lakes, US), according to the manufacturer’s instructions, and were incubated in the BD BACTEC MGIT TM automated mycobacterial detection system. After the instrument was culture-positive, the samples remained incubated at 37 °C for an additional 2 weeks to obtain more bacterial biomass. Culture on blood agar and Ziehl–Neelsen stain were performed for quality assessment.

### Data collection

The study used 152 MTB clinical isolates that were obtained from the samples stored in the NHLS Polokwane TB repository. All samples had previously been tested for MDR-TB using the GenoType MTBDRplus LPA during the diagnostic period January 2023 – December 2023. Among the isolates, 64% (*n* = 99/152) were classified as DR-TB and 35% (*n* = 53/152) were classified as drug-sensitive TB, based on prior characterisation using the GenoType MTBDRplus assay for first-line drugs (RIF and INH) following the manufacturers’ (Hain Lifescience) protocols. Demographic information associated with the collected samples was obtained from copies of laboratory request forms submitted at the time of collection. A subset of these samples, 35 out of 152 isolates, had previously undergone WGS characterisation blindly, providing essential reference data for resolving discrepancies and validating resistance-associated mutations in target genes.

### FluoroType MTBDR assay

FluoroType testing was performed using the FluoroCycler96 instrument (Hain Lifescience, Nehren, Germany). Polymerase chain reaction mixes were freshly prepared by combining the amplification mix A (AM-A) and the amplification mix B (AM-B) according to the manufacturer’s instructions. DNA extracted using the FluoroLyse protocol was then added to the PCR mix. Manufacturer-provided positive controls and negative controls prepared during the DNA extraction process were included as quality controls. To prevent photobleaching, the prepared PCR mixes were immediately loaded onto the FluoroCycler96 device.

The FluoroType analyser software automatically detects specific mutations in three target genes, validates the results of positive and negative controls and identifies DNA from the MTB complex based on the presence of a positive *rpoB* signature. The software classifies indeterminate results as ‘IND’ and unidentified mutations as ‘MUT’ (undifferentiated mutation). An ‘invalid’ result is reported if any amplification, negative or positive control fails. Heteroresistance or mixed infections are recognised and reported when the underlying mutations are successfully identified.

### Deoxyribonucleic acid extraction and whole genome sequencing

For the WGS assay, a 1.2 mL aliquot of each MGIT culture was transferred to a 1.5 mL tube containing glass beads and heat inactivated for 20 min at 80 °C in the Biosecurity Level 3 laboratory (BSL3). Heat-inactivated aliquots were centrifuged for 5 min in the Biosafety Level 2 (BSL2) laboratory to pellet MTB cells, and 0.6 mL of the supernatant was discarded. Genomic DNA was extracted from the cultured MTB isolate, as previously described,^9^ using the NucliSENS EasyMAG assay (BioMérieux, Marcyl’Étoile, France) from a concentrated 0.06 mL volume of positive culture MGIT medium. The DNA extracts were quantified using the Qubit 4 Fluorometer (Life Technologies, Carlsbad, CA, US). The quantified DNA extracts were sent for sequencing.

Whole genome sequencing was performed using the MiSeq (Illumina, United Kingdom [UK]). Library preparation was performed using the Nextera-XT library preparation kit (Illumina, UK), and sequencing was performed using the 2 × 300 base pairs (bp) MiSeq cartridge v.3 (Illumina, UK) with a target of 30 × – 50 × paired coverage (approximately 100 × coverage). CLC Genomics Workbench was used to detect RAVs within the genes *Rv0678, atpE, Rv1979c* and *pepQ* using reference mapping against the annotated reference genome H37Rv (NC00962.3) and the quality-based variant analysis tools.

### Data analysis

The results were loaded into Microsoft^®^ Excel (Microsoft Corp.) for cleaning and catergorisation. Categorical variables performance indices (true and false negative, true and false positive, accuracy, sensitivity, specificity and accuracy) were analysed using Statistical Package for Social Sciences (SPSS) version 29.0.0.0 and MedCalc version 22.026. Categorical variables were summarised using frequencies and percentages. Graphical illustrations were employed to visually represent the data. Descriptive statistics were applied, with continuous variables reported as medians and interquartile ranges to offer a thorough depiction of the dataset. The genomic data were analysed using the bioinformatics tool (CLC Genomics Workbench) to detect resistance-associated variants.

### Diagnostic performance metrics

Diagnostic performance metrics were calculated using standard definitions:

- Sensitivity: the proportion of true positives correctly identified by the test (i.e. resistant by both FluoroType MTBDR and reference standard).
- Specificity: the proportion of true negatives correctly identified by the test (i.e. sensitive by both FluoroType MTBDR and reference standard).
- Positive Predictive Value: the proportion of positive test results that were true positives.
- Negative Predictive Value: the proportion of negative test results that were true negatives.

Invalid or indeterminate results were excluded from sensitivity and specificity calculations but were reported separately to maintain transparency.

### Ethical considerations

Ethical clearance for this study was obtained from the University of Limpopo Turfloop Research Ethics Committee (TREC/1432/2024: UG). Permission to conduct the study and access laboratory samples was granted in advance by the laboratory’s ethical board. All procedures adhered to institutional requirements and were conducted in accordance with the ethical principles outlined in the Declaration of Helsinki.

## Results

### Sample demography

**FIGURE 1:**
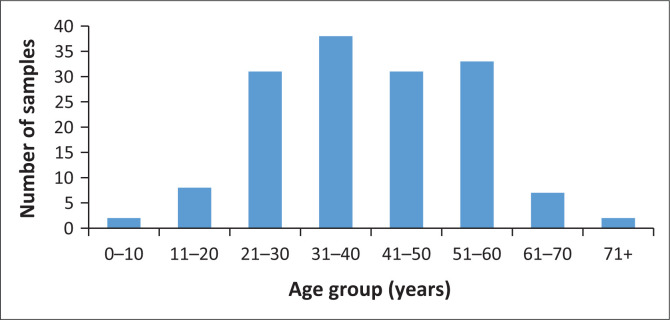
Distribution of samples per age group.

A total of 152 samples were included in the study with a median age of 40 years. Among the samples, 70 (46%) were from women and 83 (54%) were from men. Figure 1 illustrates the age distribution of the 152 MTB isolates used in the study. The highest proportion of samples came from the age group 31–40 years, with 38 samples (25%), followed by the age group 51–60 years, with 33 samples (21.7%). The age groups 21–30 years and 41–50 years each contributed 31 samples (20.4%), while the age group 11–20 years accounted for eight samples (5.3%). Smaller proportions were observed for the age groups < 1–10 years and > 71 years, each with two samples (1.3%) (Figure 1).

### Distribution of isolates according to drug resistance status

**FIGURE 2:**
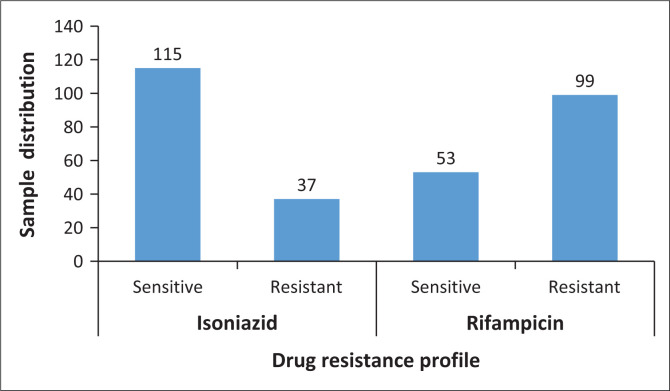
LPA drug resistance profile of the samples included for validation (*N* = 152).

Figure 2 presents the distribution of RIF- and INH-resistant isolates among the 152 samples, as characterised by the GenoType MTBDRplus assay. Based on the resistance classification of tuberculosis by LPA, 65% (*n* = 99/152) were RIF-resistant and 24% (*n* = 37/152) were INH-resistant, as previously characterised by the LPA (LPA) (Figure 2).

### Comparison of MTBDR FluoroType assay against LPA and Xpert MTB/RIF Ultra for the detection of RIF resistance

The diagnostic performance of the FluoroType MTBDR assay was evaluated against the gold standard LPA and Xpert MTB/RIF Ultra for detecting RIF resistance in 152 samples. FluoroType demonstrated a sensitivity of 96% (95% CI: 88–99) and a specificity of 100% (95% CI: 96–100) when compared to LPA, surpassing the specificity of Xpert MTB/RIF Ultra for RIF detection. Notably, six samples yielded indeterminate RIF susceptibility results, emphasising the need for assay optimisation in such cases. Six samples yielded indeterminate results for RIF susceptibility (Table 1 and Table 2).

**TABLE 1:** Diagnostic performance of FluoroType MTBDR for detection of RIF resistance, using GenoType MTBDRplus as the gold standard (*n* = 147).

FluoroType result	RIF-resistant (GenoType MTBDRplus) (*n* = 95)	RIF-sensitive (GenoType MTBDRplus) (*n* = 52)	Sensitivity		Specificity		PPV		NPV	
			%	95% CI	%	95% CI	%	95% CI	%	95% CI
RIF-resistant	91 (TP)	0 (FP)	-	-	-	-	-	-	-	-
RIF-sensitive	4 (FN)	52 (TN)	-	-	-	-	-	-	-	-
Indeterminate	4	1	-	-	-	-	-	-	-	-

**Overall**	-	-	**96**	**88–99**	**100**	**93–100**	**100**	**96–100**	**93**	**83–97**

Note: Invalid or intermediate results were excluded for the calculation of performance indices.

LPA, line probe assay; PPV, positive predictive value; NPV, negative predictive value; RIF, rifampicin; TP, true positive, TN, true negative; FP, false positive: FN, false negative.

**TABLE 2:** Diagnostic performance of FluoroType MTBDR for detection of RIF resistance, using Xpert MTB/RIF Ultra as the comparator (*n* = 151).

FluoroType result	RIF-resistant (per Xpert MTB/RIF Ultra) (*n* = 98)	RIF-sensitive (per Xpert MTB/RIF Ultra) (*n* = 47)	Sensitivity		Specificity		PPV		NPV	
			%	95% CI	%	95% CI	%	95% CI	%	95% CI
RIF-resistant	96 (TP)	3 (FP)	-	-	-	-	-	-	-	-
RIF-sensitive	2 (FN)	50 (TN)	-	-	-	-	-	-	-	-
Indeterminate	0	1	-	-	-	-	-	-	-	-

**Overall**	-	-	**95**	**88–98**	**100**	**92–100**	**100**	**96–100**	**90**	**79–96**

Note: Invalid or intermediate results were excluded for the calculation of performance indices.

LPA, line probe assay; PPV, positive predictive value; NPV, negative predictive value; RIF, rifampicin; TP, true positive; TN, true negative; FP, false positive; FN, false negative.

### Comparison of MTBDR FluoroType assay against LPA for the detection of INH resistance

Furthermore, the FluoroType MTBDR assay was evaluated for accuracy in detecting INH resistance against the LPA on 152 LPA results. The assay demonstrated an estimated sensitivity of 91% (95% CI: 77–98), indicating its ability to correctly identify INH-resistant samples and a comparatively higher specificity of 99% (95% CI: 95–100), highlighting its accuracy in identifying INH-sensitive samples. Two samples were classified as indeterminate and excluded from performance calculations to ensure the reliability of the indices. Two samples were classified as indeterminate and excluded from the calculation of performance indices (Table 3).

**TABLE 3:** Diagnostic performance of FluoroType MTBDR for detection of INH resistance, using GenoType MTBDRplus as the gold standard (*n* = 150).

FluoroType result	RIF-resistant (GenoType MTBDRplus) (*n* = 35)	RIF-sensitive (GenoType MTBDRplus) (*n* = 114)	Sensitivity		Specificity		PPV		NPV	
			%	95% CI	%	95% CI	%	95% CI	%	95% CI
INH-resistant	32 (TP)	1 (FP)	-	-	-	-	-	-	-	-
INH-sensitive	3 (FN)	114 (TN)	-	-	-	-	-	-	-	-
Indeterminate	2	0	-	-	-	-	-	-	-	-

**Overall**	-	-	**91**	**77–98**	**99**	**95–100**	**97**	**81–99**	**97**	**93–99**

Note: Invalid or intermediate results were excluded for the calculation of performance indices.

LPA, line probe assay; PPV, positive predictive value; NPV, negative predictive value; INH, isoniazid; TP, true positive; TN, true negative; FP, false positive: FN, false negative.

### Whole genome sequencing and discordant results

The WGS results were available for 35 isolates, providing crucial insights into some of the discordant cases identified in this study. Whole genome sequencing identified mutations in the rpoB gene linked to RIF resistance in 97% (*n* = 34/35) of the isolates and mutations in the *katG/inhA* genes associated with INH resistance in 57% (*n* = 20/35) of the isolates. Among the discordant results, six samples were further analysed, including two with RIF-related discrepancies and four with INH-related discrepancies. Whole genome sequencing helped resolve these conflicts by detecting specific resistance-associated mutations. However, as WGS data were only available for a subset of isolates collected prior to this study, not all discordant findings could be evaluated using WGS.

Sample A, which resulted in RIF-sensitive on Xpert MTB/RIF Ultra and indeterminate on FluoroType, was confirmed RIF-resistant by WGS, revealing the *rpoB Asp435Val* mutation.

Sample B, deemed RIF-sensitive by FluoroType but resistant by Xpert MTB/RIF Ultra, was also classified as resistant by WGS because of the *rpoB Ser450Leu* mutation.

Sample C exhibited a FluoroType-resistant result inconsistent with LPA but was confirmed resistant by WGS, detecting the *inhA c.-777C > T* mutation.

Similarly, Samples D, E and F, which were INH-sensitive on FluoroType, harboured resistance-associated mutations in the *katG* gene, as identified by WGS. These mutations included *katG Phe657_Asn660del, katG 741Arg* and *katG 18_27delACCCATTACA*, respectively. The findings underscore the utility of WGS in resolving diagnostic discrepancies and identifying mutations linked to drug resistance (Table 4).

**TABLE 4:** Discordant results for FluoroType MTBDR, Xpert MTB/RIF Ultra, LPA and whole genome sequencing and reclassification.

Discordant case	LPA	Xpert MTB/RIF Ultra	MTBDR FluoroType	MTBDR FluoroType mutation	WGS profile	WGS mutation	Mutation status
A (2143)	RIF-R	RIF-S	RIF-Indeterminate	-	RIF-R	rpoB Asp435Val	Associated with resistance
B (5548)	RIF-R	RIF-R	RIF-S	-	RIF-R	rpoB Ser450Leu	Associated with Resistance
C (6733)	INH-S	-	INH-R	InhA c-777t	INH-R	inhA c.-777C > T	Associated with resistance
D (2733)	INH-S	-	INH-S	-	INH-R	katG_Phe657_ Asn660del	Associated with resistance
E (2914)	INH-S	-	INH-S	-	INH-R	katG_*741Arg	Associated with resistance
F (7151)	INH-S	-	INH-S	-	INH-R	katG_18_27delACCCATTACA	Associated with resistance

WGS, whole genome sequencing.

### Implications of the findings

The results of this study highlight the strong diagnostic performance of FluoroType MTBDR in detecting resistance to RIF and INH, with high sensitivity and specificity, compared to LPA and Xpert MTB/RIF Ultra. Identification of specific resistance-associated mutations through WGS in discordant results further validates the accuracy of FluoroType in the detection of drug resistance.

## Discussion

This study evaluated the performance of the FluoroType MTBDR assay’s diagnostic performance in identifying RIF and INH resistance in a routine laboratory setting. The evaluation used samples from the NHLS TB repository that had previously undergone testing for MDR-TB using the GenoType MTBDRplus LPA. The study compared established routine diagnostic methods, the Xpert MTB/RIF Ultra and GenoType MTBDRplus, the gold standard method for MDR-TB detection in comparison to FlouroType MTBDR. The study revealed notable outcomes that emphasise the potential of FluoroType MTBDR as a valuable molecular diagnostic tool for MDR-TB.

This study evaluated the FluoroType MTBDR assay using DNA extracted from MDR-TB samples and compared its performance with Xpert MTB/RIF Ultra and GenoType MTBDRplus, the gold standard method for MDR-TB detection. The FluoroType MTBDR assay demonstrated comparable sensitivity and specificity in detecting resistance to RIF and INH, effectively competing with the GenoType MTBDRplus in sensitivity and matching or exceeding it in specificity.^10^

For RIF resistance detection, FluoroType achieved a specificity of 100% and a sensitivity of 92%, whereas for INH resistance detection, its sensitivity and specificity were 91% and 99%, respectively. The findings in the study underscore its accuracy, reliability and potential to improve diagnostic workflows in laboratories.^10^

Our findings are consistent with published data from a recent meta-analysis, which reported pooled sensitivity and specificity estimates of 96% and 98% for RIF resistance and 91% and 99% for INH resistance using molecular diagnostic tests.^11^ In our evaluation, the FluoroType MTBDR assay achieved 96% sensitivity and 100% specificity for RIF resistance and 91% sensitivity and 99% specificity for INH resistance, aligning closely with these pooled estimates and reinforcing the assay’s diagnostic reliability.

A study using sputum samples reported a 98% sensitivity (95% CI: 95% – 99%) for FluoroType MTBDR in detecting *M. tuberculosis* complex, with a 92% sensitivity (95% CI: 84% – 96%) in both smear-positive and smear-negative specimens.^6^ Differences in sensitivity between this study and those using MGIT cultures may reflect not only bacterial load but also differences in sample matrix and DNA quality. Although MGIT cultures typically have higher bacterial concentrations, factors such as DNA degradation, non-viable bacilli or contamination during culture processing can reduce assay performance, potentially explaining the observed differences.^12^ Additionally, as the study followed South Africa’s diagnostic algorithm, FluoroType MTBDR was not evaluated in culture-positive TB cases that were Xpert MTB/RIF-negative, leaving a gap in understanding its performance in discordant cases.^13^

Compared to WGS, FluoroType MTBDR successfully detected resistance-associated mutations in the three target genes in 88.5% (*n* = 31/35) of the sequenced isolates. Notably, FluoroType MTBDR offers several clear advantages, which include a fully automated closed system capable of testing 95 samples in a single batch. The closed system minimises contamination risks, reduces human errors and accelerates diagnostic workflows. Automation significantly reduces the risk of DNA contamination, which is typically less of a concern when working with DNA isolated from MGIT isolates that have been sub-cultured. However, DNA contamination can be a more significant issue when evaluating original untreated samples, making FluoroType MTBDR a valuable tool to minimise this risk in routine diagnostics.^8^

Unlike GenoType MTBDRplus, which requires manual interpretation of hybridisation patterns, the FluoroType results are automated, eliminating subjectivity and transcription errors. This streamlined process significantly reduces transcription errors, as results are directly imported into the laboratory information system. This automatic integration further improves the accuracy and reliability of the diagnostic workflow.^6,8^ In addition, the FluoroType assay’s streamlined setup and rapid turnaround time of approximately 3 h enhance laboratory efficiency, making it a suitable option for high-throughput facilities. FluoroType is performed in a single closed tube, preventing amplicon release and eliminating the risk of cross-contamination in the laboratory. This design also reduces the need for extensive equipment, simplifying the molecular-based drug susceptibility testing (DST) process.^6^

The FluoroType MTBDR assay can detect nearly all mutations within the promoter regions of *rpoB, katG*, and *inhA*, offering significant advantages for MDR-TB diagnostics.^6^ These promoter regions are crucial, as they harbour mutations responsible for resistance to RIF and INH, the primary drugs used to treat TB. By identifying mutations such as *rpoB Asp435Val* and *Ser450Leu* for RIF resistance and *katG* and *inhA* mutations like *inhA c.-777C > T*, the assay enables precise determination of drug resistance. This comprehensive detection capability facilitates tailored treatment regimens for MDR-TB patients, ensuring timely and accurate therapy.^.7^ This comprehensive detection capability enables a more precise identification of drug resistance mutations, facilitating the tailoring of treatment regimens to individual patients. By providing accurate and timely drug susceptibility information, FluoroType can help optimise therapeutic strategies and improve patient outcomes, particularly in cases of MDR-TB. Although indeterminate results were excluded from sensitivity and specificity calculations, they were reported separately to ensure transparency. While this exclusion aligns with standard diagnostic evaluation methods, it may lead to a slight overestimation of test performance. The presence and frequency of indeterminates remain important practical considerations in real-world implementation.

Beyond diagnostic accuracy, operational factors such as cost-effectiveness and workflow are important in assessing the utility of a new assay. Although no formal cost analysis was conducted in this study, the FluoroType MTBDR assay may offer advantages in terms of reduced hands-on time and automation, potentially lowering personnel costs and improving throughput in busy laboratories. These advantages make it a practical choice for routine implementation in resource-limited, high-burden settings. Future studies should explore cost-effectiveness and implementation outcomes in real-world settings.

This study also holds important implications for primary care. In high-burden, resource-constrained settings like rural South Africa, timely diagnosis and treatment initiation for drug-resistant TB are critical. Although the FluoroType MTBDR assay is designed for centralised laboratories, its rapid turnaround time, high-throughput processing and minimal hands-on requirements can indirectly benefit primary care by accelerating the return of results to frontline clinics. Faster detection of RIF and INH resistance supports earlier treatment adjustments, improves patient outcomes and reduces the risk of transmission in the community. By streamlining laboratory workflows and reducing diagnostic delays, the FluoroType MTBDR assay can play a key role in strengthening TB diagnostic cascades linked to primary health care services.

### Strength of the study

This study provides valuable evidence to the field of TB diagnostics, as its findings contribute to the field of rapid diagnostics, especially with respect to the detection of RIF and INH resistance, wherein the FluoroType MTBDR assay showed improved sensitivity and specificity alongside a faster turnaround time when compared to an established tool, GenoType MTBDRplus. The use of WGS as a gold standard provides strong evidence of the performance characteristics of the assay and its suitability for implementation in general clinical laboratories. These results advocate FluoroType MTBDR use as a fast and accurate alternative with a great impact on timely detection and management of MDR-TB. The study also emphasises the operational advantages of FluoroType MTBDR, such as its potential for decentralised implementation and early detection of drug-resistant TB. These results support its use as a fast and accurate alternative for MDR-TB diagnosis, with substantial implications for improving treatment initiation and TB control in high-burden, resource-limited settings, aligning closely with the needs of primary health care systems.

### Limitations

A limitation of this study is that culture-based DST was not used as the reference standard. Instead, we used the GenoType MTBDRplus assay, a WHO-endorsed molecular test, as a proxy gold standard because of its routine use and availability in the study setting. While this may introduce some bias, particularly in cases of discordant molecular results, we transparently report this limitation and contextualise the findings accordingly. Another limitation is that performance was assessed using cultured isolates rather than direct clinical specimens. While this allowed for controlled comparison, future studies should evaluate the FluoroType MTBDR assay on raw clinical samples to determine its utility in routine diagnostic settings. Also, the discrepancies observed between the FluoroType MTBDR and GenoType MTBDRplus results could be attributed to heteroresistance, which was not explored thoroughly in this study, especially given that not all samples had the corresponding sequencing data. Further exploration into heteroresistance and comprehensive validation studies across diverse settings are essential to enhance understanding of FluoroType MTBDR’s diagnostic capabilities. Despite this, the FluoroType MTBDR assay demonstrated high sensitivity to detect RIF resistance, although its sensitivity to identify INH resistance was lower. As an alternative to the WHO-recommended GenoType MTBDRplus, FluoroType offers a viable option, particularly for high-throughput laboratories. To ensure a comprehensive analysis of isolates with various mutations and mutation combinations, further validation studies are essential in different settings. It is critical to note that not all samples had corresponding sequencing data, which may have added additional valuable information to the study. Furthermore, future research should focus on evaluating the performance of this novel test in low- and middle-income countries with a high prevalence of MDR-TB.

## Conclusion

In conclusion, the FluoroType MTBDR assay represents a significant advancement in MDR-TB diagnostics, offering faster turnaround time, enhanced sensitivity, specificity and operational efficiency. With its faster turnaround time and high throughput, the FluoroType MTBDR assay presents a promising alternative to the WHO-approved MTBDRplus assay. The ability of FluoroType MTBDR to rapidly detect RIF and INH resistance makes it well-suited for integration into routine diagnostic workflows, particularly in high-burden settings and centralised laboratories, thus supporting the broader goals of primary health care systems – namely, early diagnosis, timely treatment initiation and reduced transmission at the community level.

### Recommendations

The study recommends the integration of the FluoroType MTBDR assay into MDR-TB diagnostic workflows, as it addresses limitations associated with the gold standard LPA MTBDRplus. Given its potential clinical impact on the diagnosis and treatment of DR-TB, FluoroType MTBDR should be considered for inclusion in national TB diagnostic algorithms, particularly for drug resistance screening. Additionally, large-scale multicentre validation studies utilising both clinical primary cultures and direct sputum samples are necessary to further confirm these findings. Testing in both low- and high-burden TB settings will provide a broader understanding of the test’s applicability across diverse populations and healthcare environments.
